# Solid-State Polymerization of Poly(ethylene furanoate) Biobased Polyester, I: Effect of Catalyst Type on Molecular Weight Increase

**DOI:** 10.3390/polym9110607

**Published:** 2017-11-13

**Authors:** Nejib Kasmi, Mustapha Majdoub, George Z. Papageorgiou, Dimitris S. Achilias, Dimitrios N. Bikiaris

**Affiliations:** 1 Laboratory of Polymer Chemistry and Technology, Department of Chemistry, Aristotle University of Thessaloniki, GR-541 24 Thessaloniki, Greece; nejibkasmi@gmail.com (N.K.); axilias@chem.auth.gr (D.S.A.); 2 Laboratoire des Interfaces et Matériaux Avancés, Université de Monastir, 5000 Monastir, Tunisia; mustaphamajdoub@gmail.com; 3 Chemistry Department, University of Ioannina, P.O. Box 1186, 45110 Ioannina, Greece

**Keywords:** poly(ethylene furanoate), catalysts, solid state polymerization, thermal properties

## Abstract

In this work, we report the synthesis of poly(ethylene furanoate) (PEF), catalyzed by three different catalysts, namely, titanium (IV) isopropoxide (TIS), tetrabutyltitanate (TBT), and dibutyltin (IV) oxide (DBTO), via the two-stage melt polycondensation method. Solid-state polymerization (SSP) was conducted at different reaction times (1, 2, 3.5, and 5 h) and temperatures 190, 200, and 205 °C, under vacuum. The resultant polymers were analyzed according to their intrinsic viscosity (IV), end groups (–COOH), and thermal properties, via differential scanning calorimetry. DSC results showed that the post polymerization process was favorable to enhance the melting point of the prepared PEF samples. As was expected, the intrinsic viscosity and the average molecular weight of PEF increased with the SSP time and temperature, whereas the number of carboxyl end-groups was decreased. A simple kinetic model was also developed and used to predict the time evolution of polymers IV, as well as the carboxyl and hydroxyl content of PEF during the SSP. From both the experimental measurements and the theoretical simulation results it was proved that the presence of the TIS catalyst resulted in higher transesterification kinetic rate constants and higher reaction rates. The activation energies were not much affected by the presence of different catalysts. Finally, using DBTO as a catalyst, the polyesters produced have higher crystallinity, and as a consequence, higher number of inactive carboxyl and hydroxyl groups.

## 1. Introduction

Currently, the global deficiency of non-renewable petroleum resources and the continued concern about environmental and sustainability issues stimulate the interest in biomass as alternative resource [1,2,3], which has attracted much attention, and has witnessed an incessant growth in recent years. For this reason, various research efforts have been reported on the development of sustainable polymers from the most attractive feedstock in nature, which is the biomass. In this context, much attention given to the utilization and conversion of biomass in the biorefinery industry toward the synthesis of new bio-based bifunctional monomers has recently shown a burgeoning surge. 2,5-Furandicarboxylic acid (FDCA) is such a biobased aromatic diacid monomer, which is extremely highlighted as one of the most promising chemicals, readily prepared by catalytic oxidation of 5-hydroxymethylfurfural (HMF). The latter is an obvious precursor to FDCA [4], which can be extracted from polysaccharides and sugars. Different homopolyesters derived from the renewable-based monomer (FDCA) and several diols have testified a to continuous growth in recent decade [5,6,7,8,9,10,11]. Among them, poly(ethylene furan dicarboxylate) (PEF) is the most successful biobased polyester, as it is produced from 2,5-furandicarboxylic acid (2,5-FDCA) and ethylene glycol. It is 100% biobased alternative to its commercial analog derived from petrochemical origin polyethylene terephthalate (PET) [12]. Recently, the potential progress on PEF has been intensively reviewed in two extended reviews [13,14]. This commercial polyester has continually acquired an increased interest due to its exceptional properties, such as a superior barrier performance, attractive mechanical properties [15], improved thermal stability up to approximately 320 °C [16], reduced carbon footprint [17], and its ability to formulate in films, fibers, and mostly bottles. In this context, Avantium has started, in 2010, manufacturing PEF for typical applications for fibers and packaging of water, soft drinks, and alcoholic beverages, among others, via Avantium’s YXY technology [18].

Although PEF exhibits better features compared with its analog, PET, an obstacle still currently a problem of interest for researchers, besides the undesired yellow discoloration of the resulting polyester, is the production at high molecular weight, which makes it possible to avoid the low mechanical properties of PEF, and thus, strengthen performance. This disadvantage could be associated to the type of catalysts used, which has an important role in molecular weight increase, as well as due to the decomposition of 2,5-FDCA at high reaction temperatures during melt polycondensation reactions.

A number of papers on PEF synthesis have been published involving the effect investigation of a wide range of experimental conditions during both transesterification and melt polycondensation reactions on the enhancing of its molecular weight (*M*_n_). In this context, Gruter et al. [19] described a systematic study, performed on small scale in polycondensation film reactors, on the synthesis of PEF via melt polycondensation using two different catalysts; titanium (IV) isopropoxide and butyltin (IV) tris(octoate)-tris(nonylphenyl)phosphite mixed catalyst system. Results have shown that increasing the concentration of the first catalyst over 0.4 μmol does not lead to an increase of molecular weight, whereas using higher concentration of the second catalyst system results in higher *M*_n_. The addition of tris(nonylphenyl)phosphitemay acts as a heat stabilizer, to reduce, considerably, the coloration of the resulting polyester, PEF. This study was focused mainly on the discoloration of PEF by varying the used catalyst type.

To obtain high molecular weight polyesters appropriate for several applications (e.g., bottles, films, and fiber production), an extensively-used method has been industrially applied as a third-step to polyesters, which is solid-state polymerization (SSP) performed under mild conditions. This alternative technique to the conventional melt polycondensation, involving heating the polyester in temperatures just lower from its melting point and higher than its glass transition temperature, was greatly exploited in PET [20,21,22,23,24,25,26,27] to overcome its low molecular weight.

In the case of PEF, much work has been focused on the investigation of its glass transition, thermal properties, and isothermal or non-isothermal crystallization [28,29,30,31,32,33,34,35,36,37], as well as on the synthesis and characterization of this biobased polyester [38,39,40,41]. However, contrary to expectations, very limited publications relative to the application of SSP to PEF have been reported, notwithstanding its numerous potential advantages with respect to production of high molecular weight polyester. To the best of our knowledge, to date, only three earlier studies [42,43] have been conducted on SSP of PEF as a subsequent third-stage after two-stage melt polymerization. In this context, SSP procedure was successfully applied in PEF, involving Ti(IV)-isopropoxide as catalyst, by Knoop et al. [29] aiming at increasing the polyester degree of polymerization during several days. The *M*_n_ was increased to 25,000 g/mol and then to 83,000 g/mol, respectively, after 24 and 72 h of heating at 180 °C. This study mainly tackled the preliminary crystallization investigation and its influence on the mechanical properties of high molecular weight PEF.

In addition, investigations reporting on the effect of the catalyst type on molecular weight increase of poly(ethylene furanoate), using solid-state polymerization, have not yet been reported. Therefore, the objective of the present work was to study extensively the feasibility of PEF SSP, using a series of catalysts namely; tetrabutyltitanate (TBT), titanium (IV) isopropoxide (TIS) and dibutyltin (IV) oxide (DBTO). The effect of the catalyst type, along with the influence of other two critical parameters; reaction time and temperature, on the molecular weight increase of the resulting polyester PEF was investigated in detail using both experimental measurements and a simple kinetic theoretical model.

## 2. Experimental

### 2.1. Materials

2,5-furan dicarboxylic acid (2,5-FDCA, purum 97%), ethylene glycol (99.8%), tetrabutyltitanate (TBT) (97%), titanium (IV) isopropoxide (TIS) (97%) and dibutyltin (IV) oxide (DBTO) (98%) catalysts were purchased from Aldrich Co. (Chemie GmbH, Unna, Germany). All other materials and solvents used were of analytical grade.

### 2.2. Synthesis of 2,5-Dimethylfuran-dicarboxylate (DMFD)

DMFD was prepared according to reported procedure [44], where by a reaction was carried out into a round bottom flask (500 mL) in presence of 2,5-furandicarboxylic acid (15.6 g), 200 mL of methanol anhydride and 2 mL of concentrated sulfuric acid. The mixture was refluxed for 5 h. Subsequently, the excess of methanol was removed by distillation and filtration was carried out through a disposable Teflon membrane filter (Chemie GmbH, Unna, Germany). During filtration, DMFD precipitated as white powder and then 100 mL of distilled water was added after cooling. An addition of Na_2_CO_3_ 5% *w*/*v* during stirring was used to neutralize partially the dispersion. DMFD was taken as white powder, which was collected by filtration and washed with distilled water and after drying was recrystallized using a mixture of 50/50 *v*/*v* methanol/water. According to this procedure white needles of DMFD were prepared (yield about 83%) with melting point 115 °C and purity measured with NMR 99.5%.

### 2.3. Polyester Synthesis

PEF samples were prepared through the two-stage melt polycondensation method (esterification and polycondensation) in a glass batch reactor as described in our recent work [45]. DMFD and ethylene glycol at a molar ratio of diester/diol = 1/2 were charged with 400 ppm of each catalyst (TBT, TIS, DBTO) into the reaction tube of the polyesterification apparatus. The reaction mixture was conducted under controlled argon flow for 2 h at a temperature of 160 °C, for additional 1 h at 170 °C, and finally for 1 h at 180–190 °C. This first step (transesterification stage) was considered complete after the collection of almost all the theoretical amount of methanol, which was removed from the reaction mixture by distillation and collected in a graduated cylinder. In the second stage of polycondensation, the pressure was gradually reduced to 5.0 Pa over a period of about 30 min, to remove the excess diol, to avoid excessive foaming, and to minimize oligomer sublimation, which is a potential problem during melt polycondensation. The temperature was gradually increased to 230 °C, while stirring speed was also increased to 720 rpm. The reaction kept at this temperature for 2 h. After the polycondensation reaction was completed, PEF was easily removed from the reactor, milled, and washed with methanol.

### 2.4. Solid-State Polycondensation

Solid-state polymerization (SSP) was carried out using an apparatus involving five volumetric flasks (100 mL), which were connected to a vacuum line and were immersed in a potassium nitrate/sodium nitrite thermostated bath, having a precision within ±0.5 °C. PEF (2 g) with a particle size fraction of −0.40 + 0.16 mm was placed in each one of the volumetric flasks under vacuum, stabilized beneath 3 and 4 Pa. The reaction temperature was kept constant at 190, 200, or 205 °C. The reaction flasks were taken from the bath after 1, 2, 3.5, and 5 h for analysis of the PEF sample’s intrinsic viscosity (IV), to identify the molecular weight of the polyester, as well as measuring the carboxyl end-group content (COOH).

### 2.5. Polyester Characterization

#### 2.5.1. Intrinsic Viscosity Measurement

For intrinsic viscosity [η] measurements, PEF samples (1 wt %) have been dissolved in phenol/tetrachloroethane (60:40 *w*/*w*) mixture at 90 °C, and their flow time was measured using an Ubbelohde viscometer (Schott Gerate GMBH, Hofheim, Germany) at 25 °C. The [η] values were calculated using the following Solomon-Ciuta equation:

[η] = [2{*t*/*t*_0_ − ln(*t*/*t*_0_) − 1}]^1/2^/*c* (1)

where *c* is the concentration of the solution; *t*, the flow time of solution; and *t*_0_ the flow time of pure solvent. For each sample, the measurement was repeated three times to ensure the accuracy of the results, and the average value was calculated.

#### 2.5.2. Molecular Weight

The number average molecular weight (${\bar{M}}_{n}$) of the PEF samples was calculated from the intrinsic viscosity [η] values, using the Berkowitz equation [46], as was modified in our previous work [47]:

(2) $${\bar{M}}_{n}=3.29\times 10^{4}\;{\left[\eta \right]}^{1.54}$$

#### 2.5.3. Wide Angle X-ray Diffraction Patterns (WAXD)

X-ray diffraction patterns of the PEF samples were performed in the wide-angle region by using a MiniFlex II XRD system from Rigaku Co. (Tokyo, Japan), with CuKα radiation (λ = 0.154 nm) in the angle (2θ) range from 5° to 65°, at steps of 0.05° and counting time of 5 s per step.

#### 2.5.4. End-Group Analysis

Carboxyl end-group content (C.C.) of the PEF polyesters was determined according to Pohl’s method, by titrating a solution of the polyester in benzyl alcohol/chloroform mixture. NaOH solution in benzyl alcohol was used as standard solution, and phenol red as indicator [48]. For each sample, three different measurements were carried out and the average value was calculated.

#### 2.5.5. Differential Scanning Calorimetry (DSC)

Differential scanning calorimetry (DSC) study of PEF was performed using a Perkin-Elmer, Pyris Diamond DSC differential scanning calorimeter (Perkin–Elmer, Waltham, MA, USA), calibrated with high purity metal standards. For each measurement, a sample of 7 ± 0.1 mg was sealed in aluminum pans, and was then heated from 30 to 240 °C at a heating rate of 20 °C/min under nitrogen flow (50 mL/min). The glass transition temperature (*T*_g_), the melting temperature (*T*_m_), and the heat of fusion (Δ*H*_m_) of the PEF samples were determined from these scans.

## 3. Modeling the PEF SSP Kinetics

### 3.1. Reaction Mechanism

The reactions taking place during SSP of PEF include polycondensation/transesterification, esterification, thermal degradation, and side reactions of vinyl end-groups [20], and they are illustrated in the following Equations (3)–(6). In these equations, *k*_1_, *K*_1_ and *k*_2_, *K*_2_ denote the forward and equilibrium rate constants of transesterification and esterification reaction, respectively, *k*_d_ and *k*_v_ refer to the kinetic rate constants of the degradation and polycondensation of vinyl end-group reactions, which are considered one way.

Polycondensation/transesterification
(3)
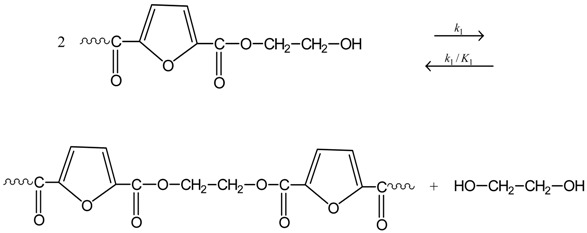

Esterification(4)
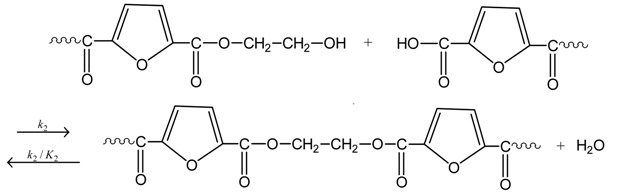

Thermal degradation(5)
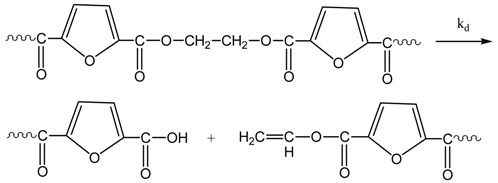

Polycondensation of vinyl end-groups(6)
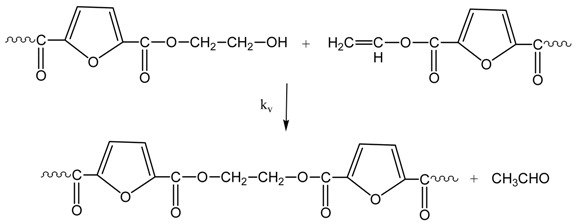


The molecular weight of the polymer is increased by two reactions: in the first, Equation (3), two hydroxyl end-groups react, and ethylene glycol is produced. In the second, Equation (4), a carboxyl end-group reacts with a hydroxyl, and water is released as by-product. In contrast, when thermal degradation takes place (Equation (5)), the molecular weight of the polyester can be decreased from the cleavage of an ester bond in the macromolecular chain, generating a vinyl ester end-group and a carboxyl end-group. In addition, acetaldehyde may be released from the side reaction of a vinyl ester end-group with a hydroxyl end-group, resulting also in an increase of the molecular weight (Equation (6)). The overall reaction rate is influenced by a combination of several factors, including intrinsic reaction kinetics, change of polymer degree of crystallization, and diffusional limitations of the reactive end-groups, and of the desorbing volatile by-products (i.e., glycol and water) [49].

### 3.2. Simplified Mathematical Model

The problem of modelling the SSP kinetics is complicated, since, besides chemical kinetics, describing the rate of change of the concentration of the species as a function of time, diffusion phenomena should be incorporated, which results in additional variation with the distance from the interface [20]. Thus, two independent variables are introduced, resulting in a set of partial differential equations that should be solved, including several kinetic, diffusional, and crystallization parameters [50]. Using such complicated models to simulate a few experimental data points is out of any physical meaning. Since in this investigation, only five data points were measured at each experimental condition, a simple kinetic model was adopted after Agarwal and co-workers [51,52]. This approach was originally developed for the solid-state polycondensation of PET, and successfully applied by our group in modelling the SSP of PET with several nanoadditives, as well as of PEF with nanoadditives [53,54,55,56].

In order to develop the mathematical model, several assumptions were made, including the following:

- All kinetic rate constants are considered independent of polymer chain length (only end-group reactivity is considered).
- Backward reactions in Equations (3) and (4) are eliminated, due to the fast removal of the water and ethylene glycol, produced in the reaction mixture, caused by the application of high vacuum (beneath 3 and 4 Pa).
- Due to the performance of the polycondensation at relatively low temperatures (i.e., 190–205 °C), no side reactions for the formation of acetaldehyde or thermal degradation are considered (Equations (5) and (6) are eliminated).
- Diffusional limitations on account of desorbing volatile species are neglected.

Then, the rate of change of hydroxyl, [OH] and carboxyl, [COOH] end-groups can be described by the following expressions [51,52]:

(7) $$\frac{d{\left[OH\right]}_{t}}{dt}=-2k_{1}{\left[OH\right]}_{t}^{2}-k_{2}{\left[COOH\right]}_{t}{\left[OH\right]}_{t}$$

(8) $$\frac{d{\left[\mathrm{COOH}\right]}_{t}}{dt}=-k_{2}{\left[\mathrm{COOH}\right]}_{t}{\left[\mathrm{OH}\right]}_{t}$$

where [OH]*_t_* and [COOH]*_t_* denote the actual “true” hydroxyl and carboxyl end-group concentrations, respectively.

The term “actual hydroxyl and carboxyl end-groups” was introduced by Ma and Agarwal [51,52], in order to account for the slowdown in SSP kinetics at high [η] values. Accordingly, a part of the carboxyl ([COOH]) and hydroxyl end groups ([OH]) were considered to be rendered temporarily inactive (denoted as [COOH]*_i_* and [OH]*_i_*) and the actual concentration of OH and COOH in Equations (7) and (8) can be expressed as:

[OH]*_t_* = [OH] − [OH]*_i_* (9)

[COOH]*_t_* = [COOH] − [COOH]*_i_* (10)

Moreover, the number average molecular weight is expressed as

(11) $${\bar{M}}_{n}=\frac{2}{\left[\mathrm{COOH}]+[\mathrm{OH}\right]}$$

Equations (7) and (8), together with Equations (2) and (9)–(11), constitute a set of ordinary differential equations which can be easily solved numerically using a varying step-size Runge–Kutta method, to provide results on the variation of the intrinsic viscosity and the concentration of –OH and –COOH end-groups as a function of time. Four adjustable parameters, namely *k*_1_, *k*_2_, [OH]*_i_*, and [COOH]*_i_*, are estimated at each experimental condition by simultaneous fitting of the values of IV, [OH], and [COOH] to the experimental data points as a function of time.

## 4. Results and Discussion

### 4.1. Synthesis and Characterization of PEF Samples

**Figure 1:**
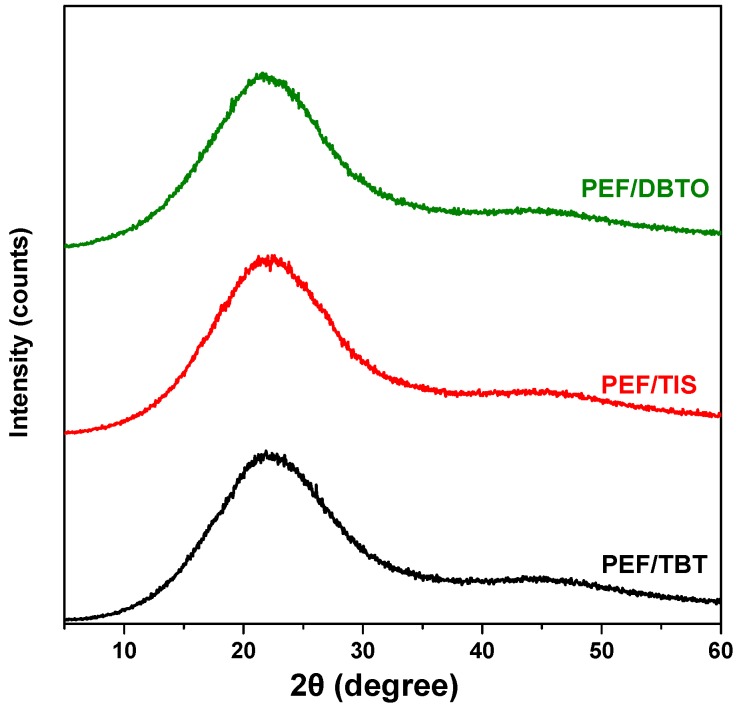
WAXD patterns of as the prepared poly(ethylene furanoate) (PEF) samples after melt polycondensation using different catalysts (TBT, TIS, and DBTO).

PEF polyester samples, with different intrinsic viscosity of 0.30, 0.31, and 0.38 dL/g, were first synthesized by melt polycondensation using three different catalysts TIS, DBTO, and TBT, respectively. The obtained polyesters were completely amorphous as can be seen from their recorded XRD patterns (Figure 1). Then, SSP was applied on the resulting samples at temperatures 190, 200 and 205 °C for 1, 2, 3.5 and 5 h. Before SSP all polyesters were crystallized for 6 h at 170 °C in an inert atmosphere.

### 4.2. Kinetic Study of the Solid-State Polymerization of PEF

**Figure 2:**
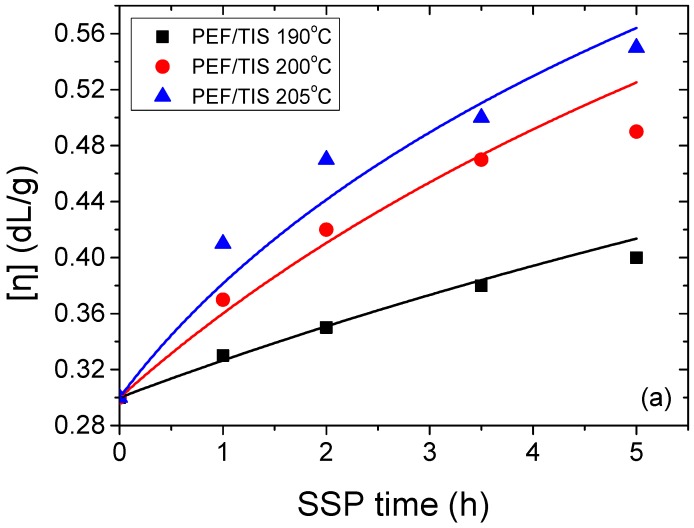

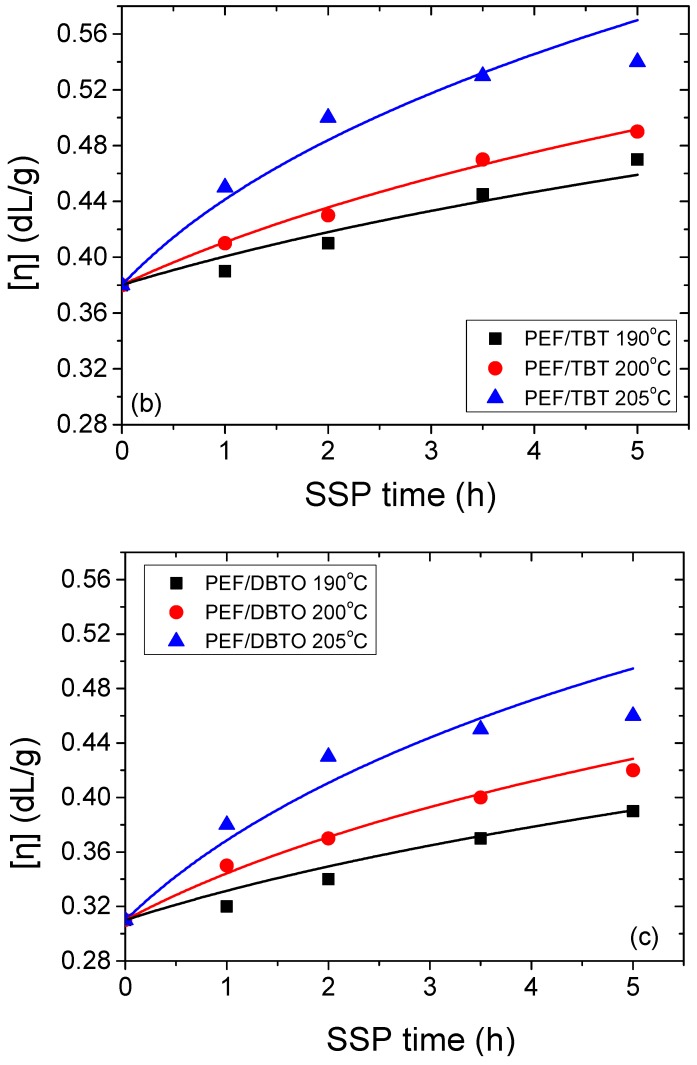
Variation of the intrinsic viscosity with time during solid-state polymerization (SSP) of PEF using different catalysts; TIS (**a**), TBT (**b**), and DBTO (**c**) at different temperatures. Continuous lines represent the theoretical kinetic model simulation results.

SSP is a very promising and well-known technique for increasing the molecular weight of polymers synthesized by melt polycondensation. Although much work has focused on SSP of PET, very little has been reported in literature on the SSP of PEF, and the effect of catalyst type, along with the effect of reaction temperature with SSP time, have never been investigated before. In this work, the intrinsic viscosity (IV) of all prepared polyester samples was measured at different reaction times and temperatures. As shown in Figure 2, an increase in the IV values with increasing reaction temperature and time was observed for all PEF samples during SSP, whatever the used catalyst type. This is mainly attributed to the solid-state polymerization temperature rising, allowing, consequently, an enhancement of the SSP rate by increasing the end-group mobility, end-group reactivity, and diffusion rate effectiveness of the by-products (e.g., ethylene glycol and water). The IV of PEF/TIS starts from 0.30 dL/g and increases to 0.40, 0.49, and 0.55 dL/g after 5 h of SSP at 190, 200, and 205 °C, respectively. For PEF/TBT, an increase in the IV was observed, starting from an initial value of 0.38 dL/g to 0.48, 0.49, and 0.54 dL/g after 5 h of SSP at 190, 200, and 205 °C, respectively. Whereas, for PEF samples using DBTO as catalyst, the IV increased from the initial value of 0.31 dL/g to 0.40, 0.41, and 0.46 dL/g after 5 h of SSP at 190, 200, and 205 °C, respectively. The viscosity values of final polymer indicate an overall activity of the three used catalysts in the SSP, but with a lower activity for DBTO compared with TBT and TIS.

For the samples produced at 190 °C, which is about 20–30 °C lower than the melting point of PEF, the lowest increase of IV was measured, regardless of the catalyst type. This effect could be explained by the slowness of the transesterification and esterification reactions as these two latter are controlled by the diffusion of byproducts produced, which is much slower at low SSP temperatures [26,27]. This is what was shown and confirmed by increasing the SSP temperature to 200 °C, whereby a slight increase in the IV was observed compared to those at 190 °C for PEF/TBT and PEF/DBTO, whereas a significantly higher increase IV was detected for PEF/TIS. At the higher reaction temperature of 205 °C, for all PEF samples whatever the catalyst used, the IV increased rapidly within the first 2 h of SSP, while it increased slightly slower within the first 3.5 h of SSP at 200 °C. This may imply that is due to the higher mobility of the molecular chains of PEF at temperature close to its melting point, and thus, the carboxyl end-groups react more easily with hydroxyl end groups, joining the macromolecular chains, and subsequently increasing the molecular weight of the polyester. Compared with previous work in the literature on PEF SSP, a recent study of Hong et al. [57] has been published in which SSP was applied on PEF at 195 °C using TIS as catalyst. The IV was found to increase at a very slow rate, from an initial value of 0.60 dL/g, to 0.64 and 0.72 dL/g after 24 h and 48 h SSP, respectively. A much greater increase in IV was measured in the present paper, compared to that reported in Hong’s study. This discrepancy could be associated to the fact that, in Hong’s work, nitrogen flow was used, whereas in our study, a vacuum was applied during SSP, which could be considered as a feature allowing the increase of by-product removal.

As can be seen in Table 1, the number average molecular weight ${\bar{M}}_{n}$ of all prepared PEF polyester samples was calculated from the experimentally measured IV using Equation (2). The ${\bar{M}}_{n}$ is dependent on the SSP temperature and time, and increased progressively as both increased, as it was expected. It seems that the increase in the average molecular weight of the samples prepared using TIS as a catalyst was higher compared to other catalysts, especially at high temperatures.

**Figure 3:**
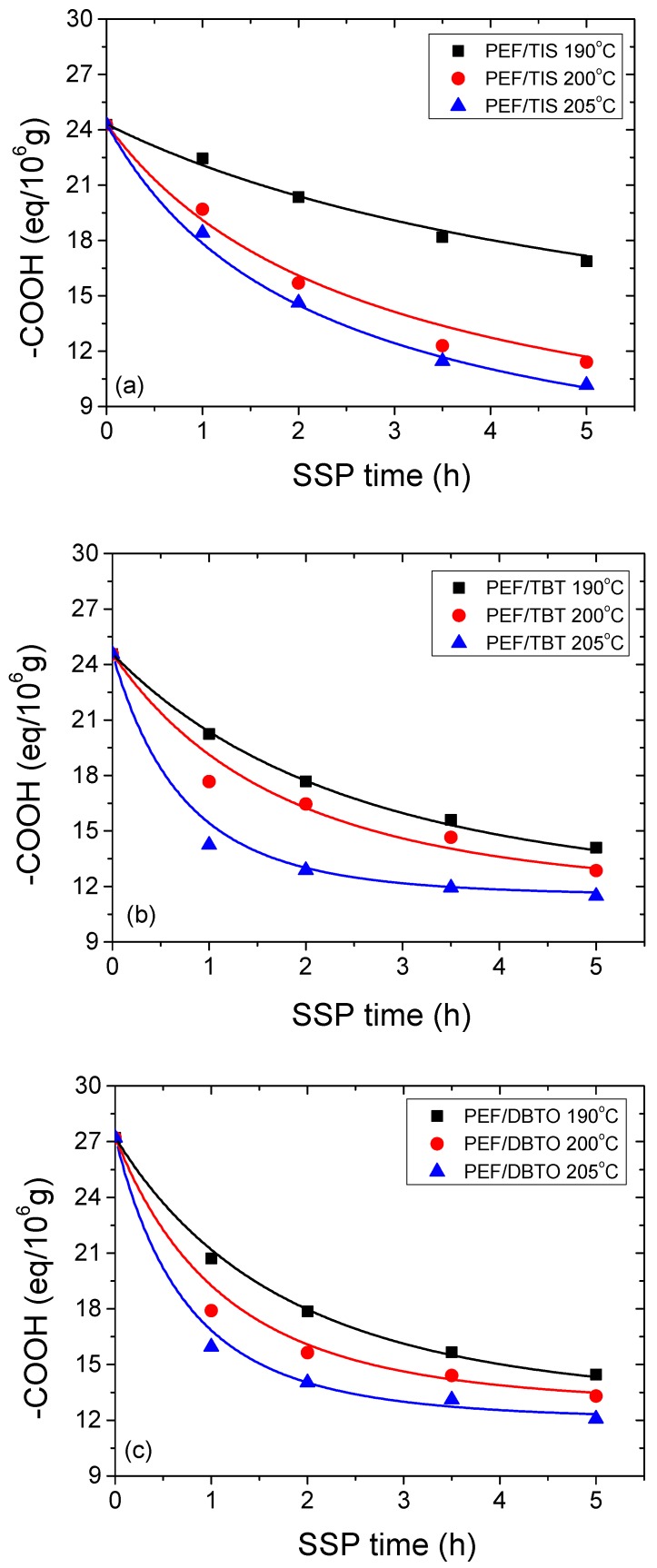
Variation of carboxyl end groups with time during PEF/TIS (**a**), PEF/TBT (**b**), and PEF/DBTO (**c**) SSP at different temperatures. Continuous lines represent the theoretical kinetic model simulation results.

Another crucial parameter usually measured to clarify the effect of SSP time and temperature on PEF SSP kinetics, is end-group analysis (–COOH), carried out on all samples prepared with different catalyst types. The measured carboxyl end groups of the PEF samples are depicted in Figure 3a–c. As it was anticipated, carboxyl end groups decrease continuously with increasing time at all SSP temperatures investigated, due to the esterification reaction taking place, with faster rates at SSP temperatures close to the melting points of the PEF polyester (205 °C). For PEF/TIS, at low SSP temperature (190 °C), the evolution of the end-group concentrations decreases slowly, and nearly linearly as a function of SSP time, whereas for the others two samples at 200 and 205 °C, the –COOH still decreases slowly, but at a higher rate. In contrast, –COOH content of the PEF/TBT and PEF/DBTO samples, regardless of the SSP temperature, greatly decrease in the first 2 h, while afterwards, the decrease of the carboxyl end groups still proceeds, but at a very slow rate. As illustrated in Figure 3, regardless of the catalyst type used, all PEF samples have a relative small content of carboxyl end groups. The latter is due to their synthesis pathway being performed by melt polycondensation method, in which the diol ethylene glycol was slightly used in excess in relation to the DMFD. Hence, the formed bis-hydroxyethylenefuranoate at the first stage of transesterification was polycondensed at the second stage to prepare PEF. Thus, it is expected that all macromolecular chains should have only hydroxyl end-groups and not carboxyl end-groups, which was the case. The existence of the latter can be explained by the decomposition reactions that are proceeding during melt polycondensation process, giving carboxyl and vinyl end-groups (Equation (5)), and some of the carboxyl ends are holdovers from the direct-esterification stage. This resulting product was also obtained in the case of PET [22] because of the thermal degradation, which plays a significant role at high temperature reaction. As the SSP proceeds, the esterification reactions are carried out in a greater extent, resulting in the reduction of the –COOH concentration, and consequently, an increase in the molecular weight of PEF was obtained (Equation (4)). This decrease is in agreement with the corresponding increase of intrinsic viscosity values at all SSP temperatures. In this connection, applying the –COOH measured values and the estimated number average molecular weight exhibited in Table 1 obtained from IV, the hydroxyl end-groups were calculated using Equation (11).

**Figure 4:**
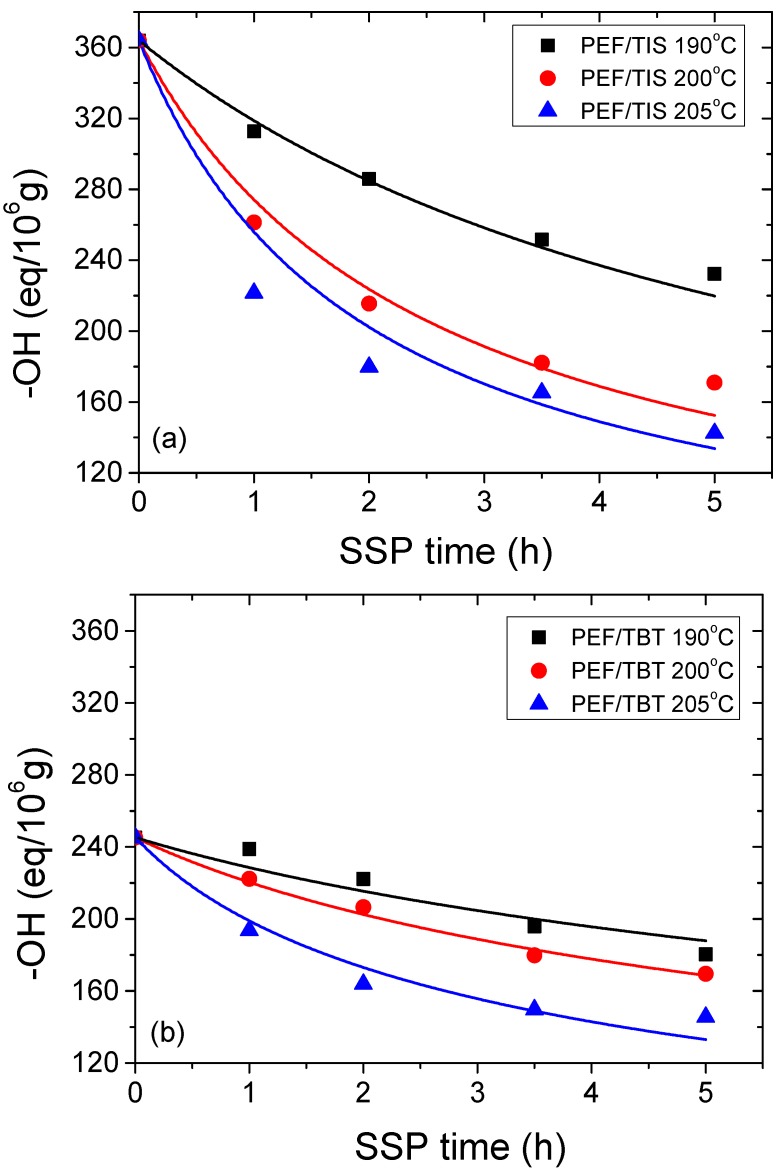

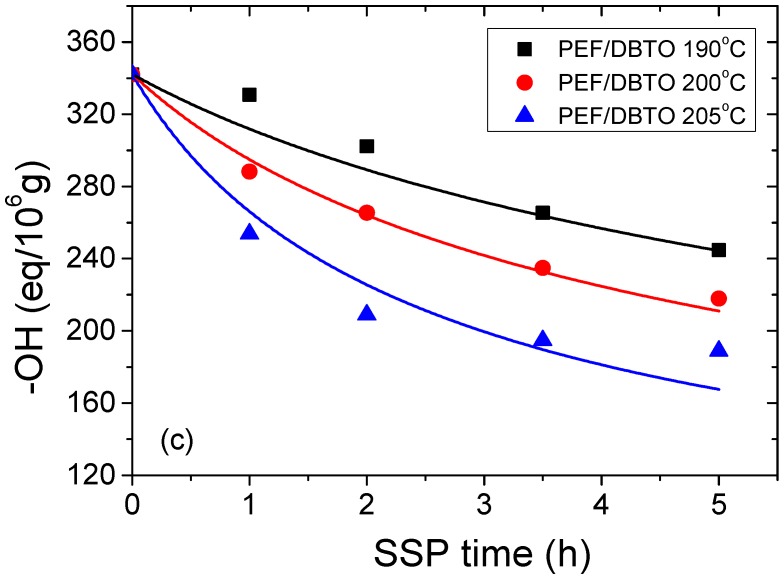
Variation of hydroxyl end groups with time during PEF/TIS (**a**), PEF/TBT (**b**), and PEF/DBTO (**c**) SSP at different temperatures. Continuous lines represent the theoretical kinetic model simulation results.

The calculated –OH content for all PEF samples using different catalysts are presented in Figure 4a–c. As can be seen in these diagrams, at low SSP temperature (190 °C), a rather small reduction in –OH groups was observed for PEF/TBT and PEF/DBTO during the first 2 h of SSP, followed by a much faster drop after 3.5 h of SSP. The slowdown at the beginning, as well as the rapid decrease rate at 3.5 h of SSP in hydroxyl content, is in total agreement with the corresponding IV values, in which a very small variation in MW during the first 2 h SSP was found, whereas a significant increase in MW was reported after 3.5 h of reaction. For PEF/DBTO, PEF/TBT, and PEF/TIS, at the higher SSP temperature of 205 °C, an initial brutal reduction of –OH groups was exhibited during the first 2 h of SSP, while afterwards, hydroxyl-group concentrations did not change much with the SSP time. This trend is also obvious at lower temperature (200 °C), but with a slower decrease rate of –OH. At all SSP temperatures and times investigated, PEF samples prepared through DBTO catalyst exhibited much higher hydroxyl end-groups compared to the two other samples, PEF/TBT and PEF/DBTO, which are very close to each other. This high difference is rather due to the rather low average molecular weight of the polyesters synthesized using DBTO. All above evidence points convincingly to the retarding effect of the catalyst DBTO on the molecular weight increase of PEF during SSP. It can therefore be stated that DBTO has lower reactivity compared to the other two catalysts, TIS and TBT, which have a similar overall behavior.

Furthermore, in order to study the kinetic mechanism in depth, and estimate appropriate rate constants, the theoretical kinetic model presented in Section 3 was employed. Differential Equations (7) and (8) were solved numerically, together with Equations (2) and (9)–(11), to provide the variation of intrinsic viscosity, as well as the concentration of hydroxyl and carboxyl end-groups as a function of SSP time. Parameters *k*_1_, *k*_2_, [OH]*_i_*, and [COOH]*_i_* were estimated from fitting of the experimental data presented in Figure 2, Figure 3 and Figure 4 for PEF prepared using different catalysts at all temperatures. The best fit value parameters are included in Table 2. Results of the theoretical simulation curves are included as continuous lines in the aforementioned figures. As it can be seen, theoretical simulation curves follow very well the experimental data points at all different experimental conditions investigated, including temperatures and catalyst used. Slight discrepancies could be attributed to the assumptions made, when developing the simple kinetic model.

From an inspection of the values reported in Table 2, it was observed that the transesterification rate constant, *k*_1_, was lower compared to the corresponding esterification rate constant, *k*_2_. Moreover, the *k*_1_ values of PEF/TIS are always higher than the corresponding for PEF/TBT and PEF/DBTO. This is a first indication that the polycondensation reaction is facilitated by the presence of TIS catalyst. The inverse was observed for the esterification rate constant, *k*_2_. In Table 1, a higher average molecular weight value was measured when using TBT as a catalyst, though one should have in mind that, in this case, the SSP started with samples having, from the beginning, higher average molecular weight.

In addition, from Table 2, it was estimated that the best fit value for the hydroxyl, [OH]*_i_*, and carboxyl inactive groups, [COOH]*_i_*, meaning those which are inaccessible to react, are always high in PEF/DBTO compared to PEF/TIS and PEF/TBT, and reduce with increasing temperature. This is attributed to the higher degree of crystallinity that these polyesters exhibit, as it will be illustrated in the next section. Higher crystallinity induces reduction in the chain mobility, which becomes more restricted, as well as hindering of the diffusion rate of reaction by-products (water and ethylene glycol) by imposing a higher degree of resistance to mass transfer. Moreover, higher temperatures facilitate the diffusion and the chain mobility, resulting in lower values of the inactive hydroxyl groups [OH]*_i_*.

**Figure 5:**
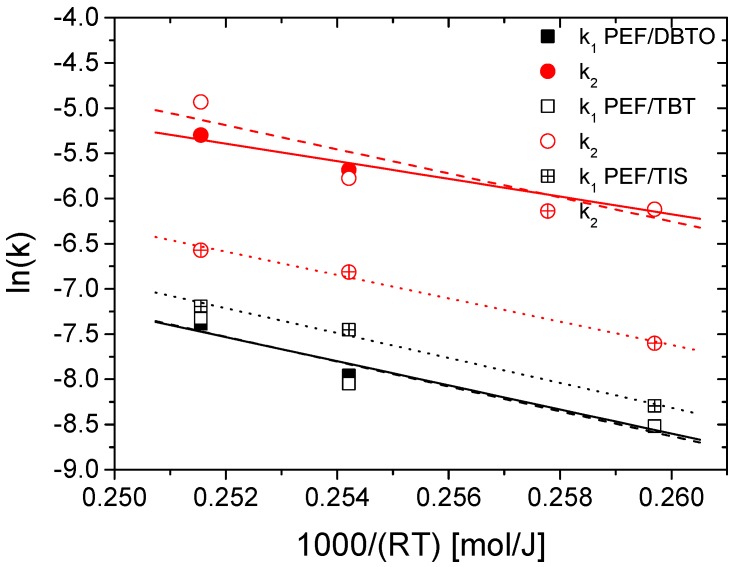
Arrhenius type plots to estimate the activation energy of the polycondensation and esterification reactions for PEF prepared by different catalysts.

As was expected, the values of all kinetic rate constants increase with SSP temperature. As the temperature is increased, the mobility and activity of the chain ends are also increased, leading to increased forward reaction rate constants. Therefore, both kinetic rate constants were correlated with temperature using an Arrhenius type expression. Results are illustrated in Figure 5. Good straight lines were obtained in most experiments with a correlation coefficient greater than 0.95. From the slope of these straight lines, the activation energies for the transesterification, *E*_1_, and esterification, *E*_2_, reactions were determined (Table 3). *E*_1_ was estimated to be near equal to 137 kJ/mol, not statistically different in all catalysts used. *E*_2_ was estimated at lower values compared to *E*_1_. From Table 3, it should be pointed that estimation of *E*_2_ for the PEF/TBT presents a rather low correlation coefficient, represented by a large error in the estimation. This could be attributed to errors in the corresponding experimental measurements. In addition, the slightly higher *E*_2_ for this system compared to other catalysts could be considered as rather artificial, since the error in this estimation is relatively high.

**Figure 6:**
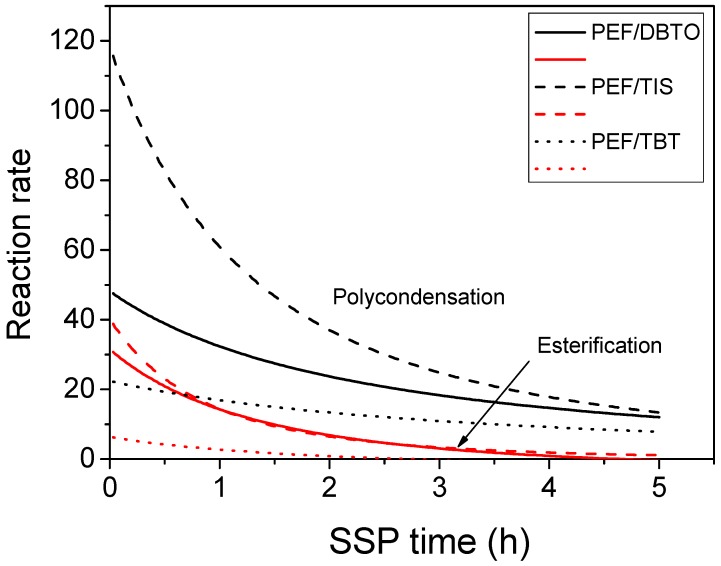
Variation of the polycondensation and esterification reaction rates with SSP time estimated during SSP of PEF/TIS, PEF/TBT, and PEF/DBTO at 200 °C.

In order to have a clear view on the effect of the catalyst type on the SSP of PEF, the reaction rates of the polycondensation (transesterification) and esterification reactions were estimated from Equations (3) and (4), i.e., as 2*k*_1_[OH]_t_^2^ and *k*_2_[COOH]_t_[OH]_t_, respectively. Results on the variation of both reaction rates with time appear in Figure 6. Initially it was observed that the transesterification reaction rate is always much higher compared to esterification. This finding is mainly due to the much higher concentration of hydroxyl end-groups during the whole reaction, compared to the carboxyl end-groups. The reaction rates are very high at the beginning of the reactions, whereas they decrease with time following the decrease in the concentration of the characteristic end groups. Both rates reach a plateau after almost 3 h of SSP time. Finally, it was clear that the polycondensation reaction rate obtained when using TIS as a catalyst was much higher compared to the other catalysts used, followed by PEF/DBTO and PEF/TBT.

### 4.3. Thermal Analysis of PEF Samples Prepared by Solid-State Polymerization

**Figure 7:**
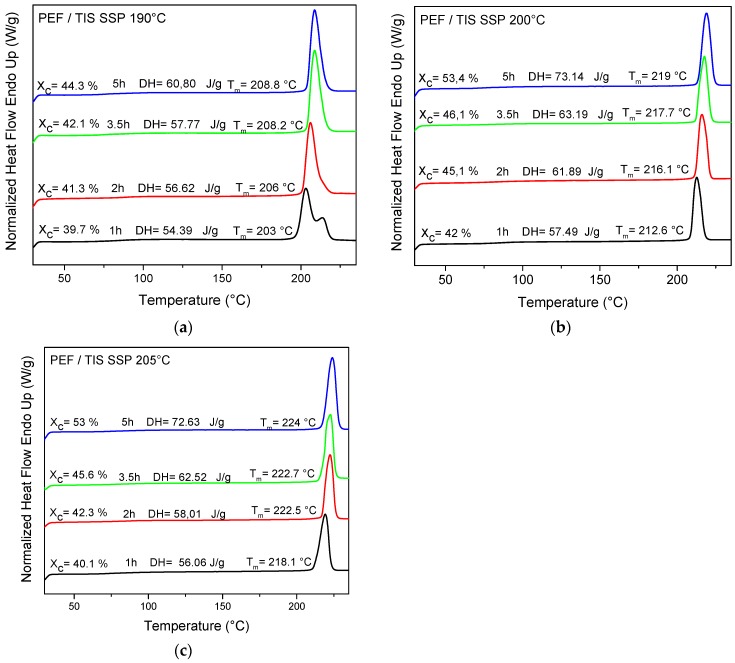
DSC thermograms of PEF/TIS samples prepared after SSP at different temperatures and times: (**a**) 190 °C, (**b**) 200 °C and (**c**) 205 °C.

The thermal properties of SSP PEF were found to be improved after the SSP process. The melting behavior of the polyester samples at different reaction times and temperatures are depicted in Figure 7 as well as in Figures S1 and S2 in the Supporting Information. For all samples, the endothermic melting peaks were shifted to higher temperatures (increased *T*_m_) with increasing of either the SSP temperature or the SSP time, and with concomitant increasing of the crystallinity. The increase of sharpness of the melting peaks, as well as the increase in the melting points, is attributed to the increased molecular weight of the polyester produced during SSP. Double-melting peaks were detected for PEF/TBT and PEF/TIS at the low SSP temperature of 190 °C processed at *t*_ssp_ 1 h. This is very usual for an SSP process, and due to the annealing process, crystals with different sizes and perfections can be formed [47,53,54,55,56]. Increasing the SSP time at the same SSP temperature, both of these high-temperature peaks at 214.6 °C disappeared, and only one endotherm is present. The dual melting endotherms areas signed to the melting–recrystallization process involving a part of the crystallites in the sample. This phenomenon is commonly observed during SSP of some alipharomatic polyesters, such as PET [58,59,60]. The recrystallization occurs through the reorganization of molecular chains, whereas in the shorter ones, having high mobility results in an easier and faster reorganization than that of long chains [61].

**Figure 8:**
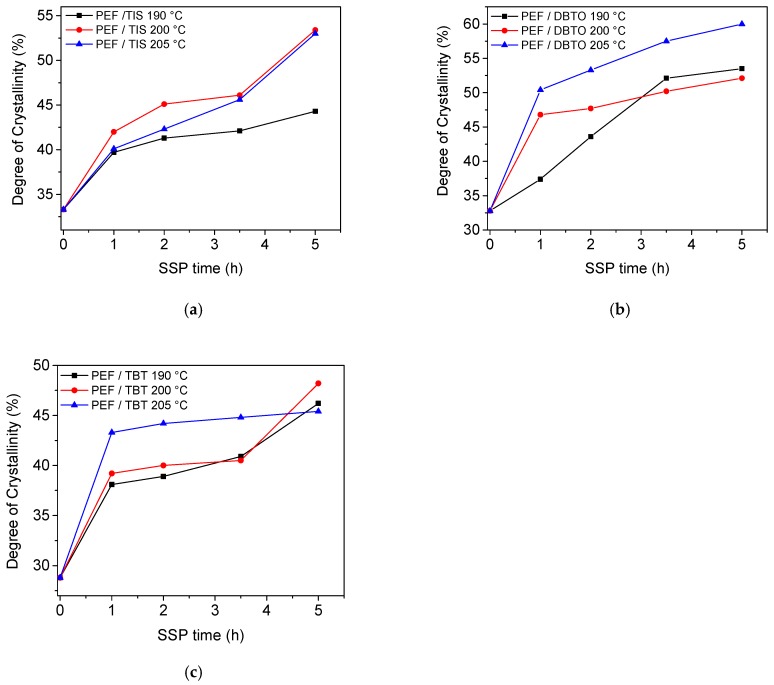
Effect of SSP time and temperature on the evolution of the degree of crystallinity of PEF samples using different catalysts: (**a**) PEF/TIS, (**b**) PEF/DBTO, (**c**) PEF/TBT.

The degree of crystallinity values (*X*_c_) for all SSP PEF samples, presented in Table 4, were calculated from measured melting enthalpy (Δ*H*_m_) using the heat of fusion value for the pure crystalline PEF found in a previous study to be about 137 J·g^−1^ [48]. PEF/TBT and PEF/TIS have almost similar *X*_c_ values, with a difference average of more or less 2%, while PEF/DBTO exhibits much higher *X*_c_ values compared with the latter two, in which the highest increase was found at 205 °C. Since SSP proceeds in the amorphous regions of the semi crystalline polymer, where end groups are excluded from crystalline regions, it can be deduced that the slowly increasing molecular weight rate/IV values of PEF/DBTO, when compared with the others two samples, is due to its highest degree of crystallinity, as illustrated in Figure 8. The latter induces, on the one hand, a reduction in the chain mobility, which becomes more restricted, and on the other hand, the diffusion rate of reaction by-products (water and ethylene glycol) may be more hindered by imposing a higher degree of resistance to mass transfer. This explanation is in good agreement with the IV/*M*_n_ values obtained in this study.

## 5. Conclusions

The effect of different catalysts on the SSP kinetics of PEF was investigated at several temperatures, both experimentally and using a simple kinetic model. As it was expected, the intrinsic viscosity and the average molecular weight of PEF increased with the SSP time and temperature. This is because the elimination of formed byproducts during both esterification and transesterification reactions that are taking place during SSP, are diffusion controlled. A simple kinetic model was also developed, and used to predict the time evolution of polymer’s IV, as well as the carboxyl and hydroxyl content during the SSP of PEF. From both the experimental measurements and the theoretical simulation results, it was proven that the presence of the TIS catalyst resulted in higher transesterification kinetic rate constants and higher reaction rates. The activation energies were not much affected by the presence of a different catalyst. Furthermore, using DBTO as a catalyst results in polyesters having higher crystallinity, and as a consequence, in higher inactive carboxyl and hydroxyl groups.

## Figures and Tables

**Table 1:** Numberaverage molecular weights (${\bar{M}}_{n}$ , g/mol) of PEF polyester using different catalysts obtained after SSP at different temperatures and times. The value includes in parenthesis is the corresponding number average degree of polymerization.

Temperature (°C)	SSP Time (h)	PEF/TIS	PEF/TBT	PEF/DBTO
	0	5152 (28)	7414 (40)	5419 (29)
190	1	5966 (32)	7717 (42)	5690 (31)
	2	7116 (38)	8335 (45)	5966 (32)
	3.5	7414 (40)	9950 (54)	7717 (42)
	5	8024 (43)	10,624 (58)	8024 (43)
200	1	7116 (38)	8335 (45)	6532 (35)
	2	8650 (47)	8969 (49)	7116 (38)
	3.5	10,286 (56)	10,286 (56)	8024 (43)
	5	10,967 (59)	10,967 (59)	8335 (45)
205	1	8335 (45)	9619 (52)	7414 (40)
	2	10,286 (56)	11,664 (63)	8969 (49)
	3.5	11,314 (61)	12,376 (67)	9292 (50)
	5	13,103 (71)	12,737 (69)	9950 (54)

**Table 2:** Kinetic rate constants of the transesterification and esterification reaction and concentration of temporarily inactivated OH and COOH end-groups at different polycondensation temperatures of PEF prepared by different catalysts, TIS, TBT, and DBTO.

Sample	Temp. (°C)	*k*_1_ (kg/meq)·h^−1^	*k*_2_ (kg/meq)·h^−1^	[OH]*_i_* (meq/kg)	[COOH]*_i_* (meq/kg)
PEF/TIS	190	2.5 × 10^−4^	5 × 10^−4^	47	8.0
	200	5.8 × 10^−4^	11 × 10^−4^	45	4.0
	205	7.5 × 10^−4^	14 × 10^−4^	44	3.0
PEF/TBT	190	2.0 × 10^−4^	22 × 10^−4^	60	11.5
	200	3.2 × 10^−4^	31 × 10^−4^	58	11.5
	205	6.6 × 10^−4^	72 × 10^−4^	52	11.5
PEF/DBTO	190	2.0 × 10^−4^	22 × 10^−4^	80	12.8
	200	3.5 × 10^−4^	34 × 10^−4^	78	12.6
	205	6.2 × 10^−4^	50 × 10^−4^	70	12.0

**Table 3:** Activation energies along with their standard errors and correlation coefficients of the transesterification and esterification reaction of PEF prepared by TIS, TBT, and DBTO catalysts.

Sample	*E*_1_ (kJ/mol)	*R*^2^	*E*_2_ (kJ/mol)	*R*^2^
PEF/TIS	137.6 ± 3.4	0.995	129.0 ± 3.2	0.995
PEF/TBT	137.4 ± 11.4	0.950	133.1 ± 15.1	0.907
PEF/DBTO	133.3 ± 6.9	0.979	97.5 ± 4.0	0.987

**Table 4:** Degree of crystallinity values (%) of the SSP PEF samples using different catalysts.

SSP Temperature (°C)	SSP Time (h)	PEF/TIS	PEF/TBT	PEF/DBTO
	0	33.3	28.8	32.8
190	1	39.7	38.1	37.4
	2	41.3	38.9	43.6
	3.5	42.1	40.9	52.1
	5	44.3	46.2	53.5
200	1	42.0	39.2	46.8
	2	45.1	40.0	47.7
	3.5	46.1	40.5	50.2
	5	53.4	48.2	52.1
205	1	40.1	43.3	50.4
	2	42.3	44.2	53.3
	3.5	45.6	44.8	57.5
	5	53.0	45.4	60.0

## Supplementary Materials

The following are available online at www.mdpi.com/2073-4360/9/11/607/s1, Figure S1: DSC thermograms of PEF/TBT samples prepared after SSP at different temperatures and times, Figure S2: DSC thermograms of PEF/DBTO samples prepared after SSP at different temperatures and times.
