# Supplementary material for: Solid-State Polymerization of Poly(ethylene furanoate) Biobased Polyester, I: Effect of Catalyst Type on Molecular Weight Increase
Source: Polymers (Basel). 2017 Nov 13;9(11):607. doi: 10.3390/polym9110607 (PMC6418636; doi:10.3390/polym9110607)
Supplement: Supplementary file 1 [file polymers-09-00607-s001.pdf]

polymers

MDPI

Supplementary

Solid-state polymerization of poly(ethylene furanoate) biobased polyester: Effect of catalyst type on molecular weight increase

Nejib Kasmi [1,2], Mustapha Majdoub [2], George Z. Papageorgiou [3,*], Dimitris S. Achilias [1] and Dimitrios N. Bikiaris [1,*]

[1] Laboratory of Polymer Chemistry and Technology, Department of Chemistry, Aristotle University of Thessaloniki, GR-541 24, Thessaloniki, Macedonia, Greece; nejibkasmi@gmail.com (N.K.); axilias@chem.auth.gr (D.S.A.)
[2] Laboratoire des Interfaces et Matériaux Avancés, Université de Monastir, 5000 Monastir, Tunisia ; mustaphamajdoub@gmail.com (M.M.)
[3] Chemistry Department, University of Ioannina, P.O. Box 1186, 45110 Ioannina, Greece
* Correspondence: dbic@chem.auth.gr (D.N.B.); gzpap@cc.uoi.gr (G.Z.P.); Tel.: +30-231-0997812 (D.N.B.); +30-265-1008354 (G.Z.P.)

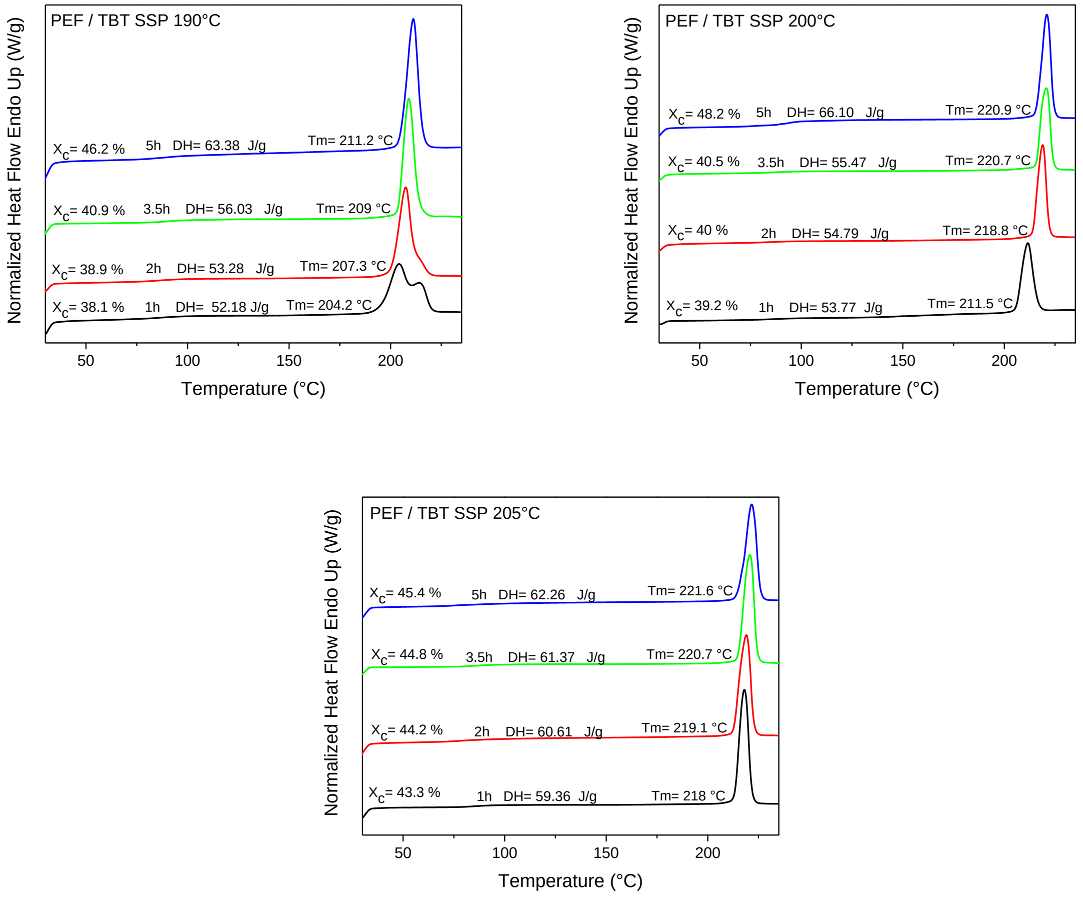

Figure S1. DSC thermograms of PEF/TBT samples prepared after SSP at different temperatures and times.

polymers

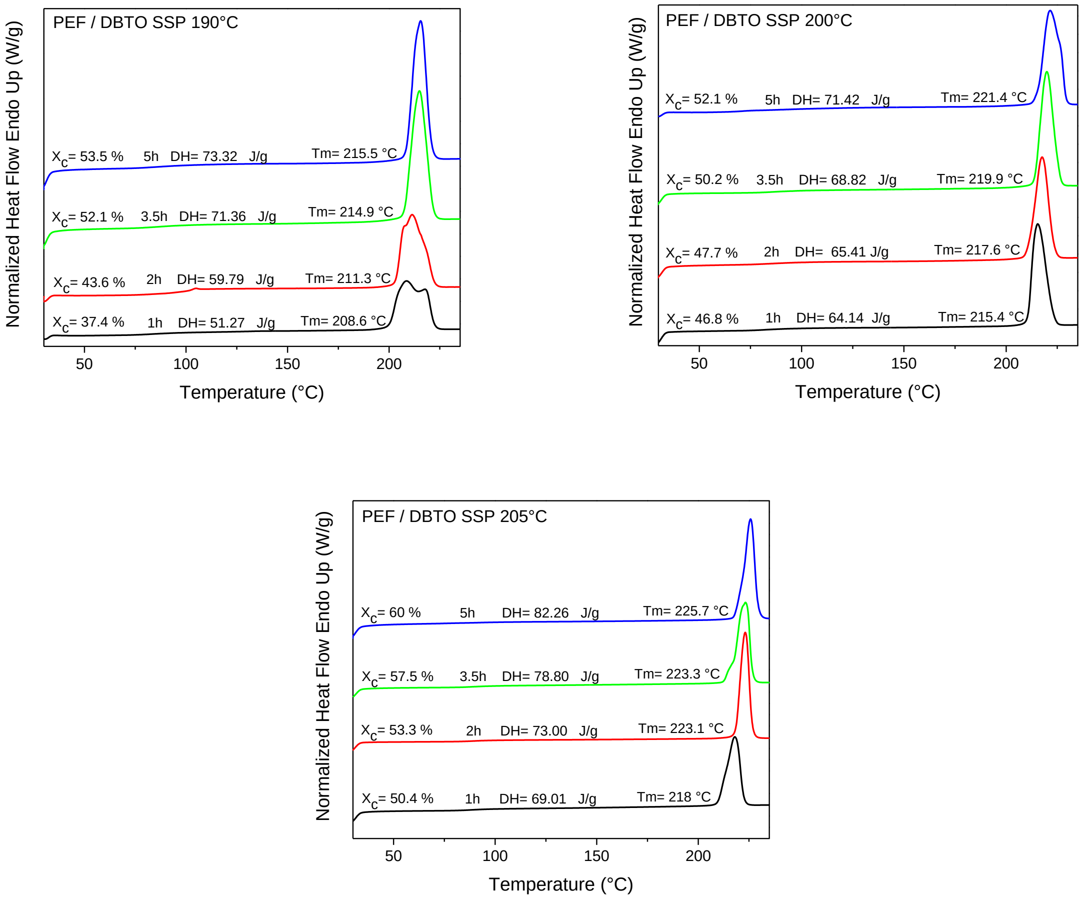

Figure S2. DSC thermograms of PEF/DBTO samples prepared after SSP at different temperatures and times.
